# Multi-Omics Integration and Network Analysis Reveal Potential Hub Genes and Genetic Mechanisms Regulating Bovine Mastitis

**DOI:** 10.3390/cimb44010023

**Published:** 2022-01-11

**Authors:** Masoumeh Naserkheil, Farzad Ghafouri, Sonia Zakizadeh, Nasrollah Pirany, Zeinab Manzari, Sholeh Ghorbani, Mohammad Hossein Banabazi, Mohammad Reza Bakhtiarizadeh, Md. Amdadul Huq, Mi Na Park, Herman W. Barkema, Deukmin Lee, Kwan-Sik Min

**Affiliations:** 1 Animal Breeding and Genetics Division, National Institute of Animal Science, Cheonan-si 31000, Korea; mina0412@korea.kr; 2 Department of Animal Science, University College of Agriculture and Natural Resources, University of Tehran, Karaj 77871-31587, Iran; farzad.ghafouri@ut.ac.ir (F.G.); manzarizeinab@alumni.ut.ac.ir (Z.M.); 3 Animal Genetics and Breeding Division, Animal Science Research Institute of Iran, Agriculture Research, Education, and Extension Organization, Karaj 31466-18361, Iran; sonia_zaki@yahoo.com (S.Z.); s.ghorbani@areeo.ac.ir (S.G.); m.banabazi@areeo.ac.ir (M.H.B.); 4 Department of Animal Science, Shahrekord University, Shahrekord 88186-34141, Iran; napirany@sku.ac.ir; 5 Department of Animal and Poultry Science, College of Aburaihan, University of Tehran, Tehran 33916-53775, Iran; mrbakhtiari@ut.ac.ir; 6 Department of Food and Nutrition, College of Biotechnology and Natural Resource, Chung-Ang University, Anseong-si 17546, Korea; amdadbge@gmail.com; 7 Department of Production Animal Health, Faculty of Veterinary Medicine, University of Calgary, Calgary, AB T2N 4N1, Canada; barkema@ucalgary.ca; 8 Department of Animal Life and Environment Sciences, Hankyong National University, Jungang-ro 327, Anseong-si 17579, Korea

**Keywords:** mastitis, transcriptome sequencing, hub genes, multi-omics data, regulatory networks, bovine

## Abstract

Mastitis, inflammation of the mammary gland, is the most prevalent disease in dairy cattle that has a potential impact on profitability and animal welfare. Specifically designed multi-omics studies can be used to prioritize candidate genes and identify biomarkers and the molecular mechanisms underlying mastitis in dairy cattle. Hence, the present study aimed to explore the genetic basis of bovine mastitis by integrating microarray and RNA-Seq data containing healthy and mastitic samples in comparative transcriptome analysis with the results of published genome-wide association studies (GWAS) using a literature mining approach. The integration of different information sources resulted in the identification of 33 common and relevant genes associated with bovine mastitis. Among these, seven genes—*CXCR1*, *HCK*, *IL1RN*, *MMP9*, *S100A9*, *GRO1*, and *SOCS3*—were identified as the hub genes (highly connected genes) for mastitis susceptibility and resistance, and were subjected to protein-protein interaction (PPI) network and gene regulatory network construction. Gene ontology annotation and enrichment analysis revealed 23, 7, and 4 GO terms related to mastitis in the biological process, molecular function, and cellular component categories, respectively. Moreover, the main metabolic-signalling pathways responsible for the regulation of immune or inflammatory responses were significantly enriched in cytokine–cytokine-receptor interaction, the IL-17 signaling pathway, viral protein interaction with cytokines and cytokine receptors, and the chemokine signaling pathway. Consequently, the identification of these genes, pathways, and their respective functions could contribute to a better understanding of the genetics and mechanisms regulating mastitis and can be considered a starting point for future studies on bovine mastitis.

## 1. Introduction

Over the last decade, advances in high-throughput genotyping and sequencing technologies [1], along with progress in developing computational methods [2], have led to a revolution towards a better understanding of the genetic architecture underlying complex traits and diseases, with exceptional depth. To date, several studies have focused on integrating different information sources (“omics” datasets) to create robust insights into complex molecular functional mechanisms by reinforcing complementary evidence from multiple levels [3,4,5]. In this regard, the results of different types of multi-layer studies have been reported, ranging from simple combinations (two different kinds of -omics data) to more comprehensive and computationally demanding ones (multiple kinds of -omics data). Incorporating two layers under a systems biology framework can involve approaches that integrate genomics and transcriptomics [6,7,8], metabolomics and transcriptomics [9,10,11], proteomics and transcriptomics [12,13], and proteomics and metabolomics analyses [14,15] to functionally characterize the interactions at the molecular level for traits of interest in humans and in livestock species.

Bovine mastitis is a common and costly disease, which has a considerable effect on the profitability of the production system, owing to its negative impacts on milk yield, quality, and reproductive performance; early culling; animal welfare issues; and the cost of treatment [16,17,18,19]. The inflammation of the mammary gland occurs in response to infection with pathogenic microorganisms or physiological and metabolic changes [20,21]. Although the heritability of mastitis is low [22], and genetic correlations between mastitis and production traits are unfavorable [23,24], genetic improvement in terms of mastitis resistance is a major breeding goal. It is also known that mastitis is highly genetically correlated with somatic cell count (SCC), which consists of macrophages, lymphocytes, and epithelial cells, and consequently, this can be used as an important indicator of udder health [25]. Furthermore, selection for correlated traits, such as reduced SCC (indicating increased mastitis resistance) could be an interesting alternative, allowing scientists to infer and comprehend the genetic and molecular mechanisms underlying these traits. In other words, the discovery of genomic regions, disease-causing genes, and biomarkers associated with mastitis is of essential importance in improving the diagnosis and treatment of the disease. In the literature, numerous studies have been carried out to identify functional candidate genes associated with mastitis based on genome-wide association studies (GWAS) [26,27,28,29,30] and transcriptome studies [6,16,31,32]. On the other hand, concordance among these studies is low, indicating difficulties in identifying reliable candidate genes for mastitis. New approaches integrating GWAS results with additional sources of information can overcome this challenge. Hence, it is worth investigating the molecular regulatory mechanisms through which mastitis can be developed. Therefore, the objective of this study was to use the integration of previously published RNA-Seq and microarray data with GWAS results to identify and prioritize potential hub genes and create reconstructions of the protein–protein interaction (PPI) and gene regulatory networks, as well as modeling of the three-dimensional hub protein structure involved in pathological processes related to mastitis in dairy cattle.

## 2. Materials and Methods

**Figure 1:**
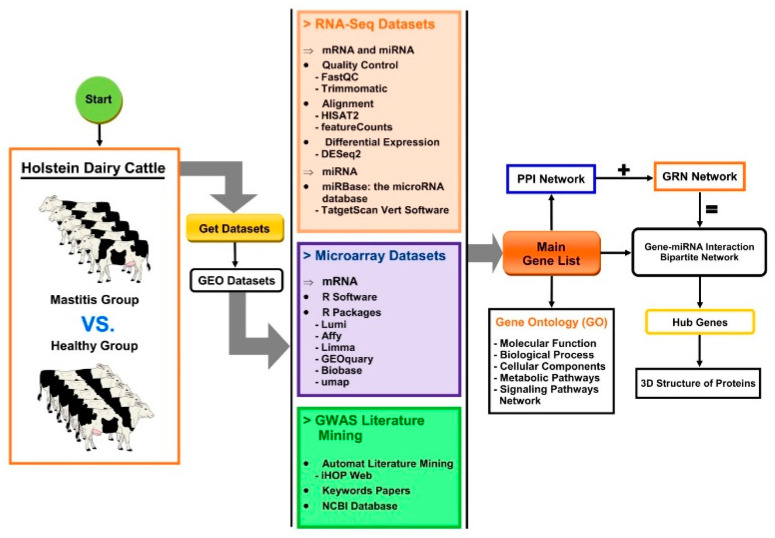
Schematic of the workflow used to reconstruct the metabolic pathways of mastitis in dairy cattle. The main gene list was prepared from RNA-Seq and microarray datasets, and literature mining. The protein–protein interaction network (PPI), gene regulatory network (GRN), and interactive bipartite network of gene–miRNA interactions were reconstructed using Cytoscape.

The overall workflow for the data collection and the analysis of relevant genes related to mastitis in dairy cattle is presented in Figure 1.

### 2.1. Data Collection

Collection and evaluation of the available data is the first step in better understanding the reconstruction of molecular networks and the biological basis in terms of the identification of candidate genes, gene regulation, interactions, protein–protein interaction (PPI), and metabolic signaling networks. In this study, the microarray and RNA-sequencing (RNA-Seq) datasets, available in the public repository of the National Center for Biotechnology Information (NCBI) Gene Expression Omnibus (GEO), were retrieved for samples of mastitis and healthy *Bos taurus* species. The accession numbers for the RNA-Seq and microarray datasets are shown in Table 1. Six Holstein cows from first to third lactations and days in milk (DIM) ranging from 7 to 236 were included in the GSE131607 dataset. All cows were kept in freestall housing at the University of California–Davis, fed a total mixed ration (TMR), and given ad libitum access to water. Two different samples were taken from each cow after diagnosis using the California Mastitis Test, one sample from the mastitic quarter (*n* = 6), and the other sample taken diagonally across from the mastitic quarter, which was confirmed as the healthy quarter (*n* = 6), based on having a somatic cell count (SCC) less than 100,000 cells/mL milk [16]. The GSE15020 and GSE15022 datasets were related to microarray analysis from a study by Mitterhuemer et al. [31]. Fifteen healthy German Holstein Frisian cows in mid-lactation (3 to 6 months postpartum) were included in the study. Quarter milk samples were collected and tested weekly before the trial to ensure that they contained <50,000 somatic cells/mL and were free of mastitis pathogens. The animals were inoculated in one quarter with *E. coli* and slaughtered after 6 h (*n* = 5) or 24 h (*n* = 5) in two different infection methods. Five cows, considered as controls, received no treatment and were slaughtered after 24 h. In total, 89 healthy and 75 diseased German Holstein cows were tested for the GSE93082 dataset. Diseases were distinguished by either systemic (extra-mammary) occurrence or those affecting the mammary gland (mastitis) to account for influences on the milk composition from local inflammatory processes. All cows were examined thoroughly by the dairy herd manager, trained staff, or a veterinarian. Healthy animals (2–4 years old, 1st to 3rd lactation, one animal 4th and one 8th lactation) which had no clinical signs of disease and no abnormalities in the udder or milk, with a reported somatic cell count less than 100,000 cells/mL were chosen as controls. Most of the control samples were taken during early lactation within 10 to 100 days postpartum. Diseased animals were in the 1st to 8th lactation period from 10 to 220 days postpartum. The milk samples of control animals or cows with extra-mammary diseases were collected and tested from one quarter or a composite milk sample (equal volumes from all 4 quarters mixed) [32]. In the GSE75379 dataset, sixteen healthy primiparous Holstein cows were inoculated with live *E. coli* into one mammary quarter at four to six weeks after parturition. The cows were housed in straw-bedded tie-stalls, where they were individually fed and given free access to water. The animals were fed using a TMR based on corn silage, minerals, and vitamins for ad libitum intake twice daily. Daily feed intake and milk yield at each milking were recorded. Prior to the start of the study period, cows were considered healthy and free of mastitis-causing pathogens based on body temperature, white blood cell count (WBC), California Mastitis Test, glutaraldehyde test, SCC, and bacteriological examinations of milk samples. Control quarters were selected based on bacteriological tests, in which quarter foremilk SCCs were <181,000 cells/mL at 24 h post-intramammary infection (IMI). Biopsy specimens of healthy and diseased udder tissue were performed 24 h post-IMI in infected and non-infected (control) mammary quarters [33].

### 2.2. Differential Gene Expression Analysis

Microarray data were pre-processed and normalized using the Lumi package [34] and the GCRMA algorithm (GeneChip Robust Multi-array Averaging) method, implemented in the Affy package in R software, to remove the variance and to prepare the data for further analysis [35]. Gene expression analysis was performed in R/Bioconductor software to screen the significant differential expression genes (DEGs) according to the comparison of the test and control data using the packages Limma [36], GEOquary [37], Biobase [38], and Umap [39].

Concerning RNA-Seq data, the quality of the raw data was assessed using FastQC software (v0.11.9) [40]. Then, based on the results of the raw data quality control, the sequences were edited to remove the adapters, PCR primers, and low-quality reads using Trimmomatic software (v0.38.0) [41]. Alignment sequences and mapping of reads were conducted on the *Bos taurus* reference genome (http://ftp.ensembl.org/pub/release-103/fasta/bos_taurus/dna/ (accessed on 20 September 2021)) using HISAT2 software (v2.1.0) [42]. For transcript quantification, featureCounts software (v2.0.1) was employed to measure the total raw counts of mapped reads [43]. DESeq2 software (v2.11.40.6) was applied for the measurement of final differences in gene expression [44]. In addition to DEGs, the identification of miRNAs was also performed in the RNA-Seq datasets, simultaneously. Finally, the threshold for statistical significance of the differential expression of each gene was obtained with the criteria of a |log fold-change (FC)| ≥ 2.0 and a false discovery rate (FDR) ≤ 0.05 in accession numbers related to microarray and RNA-Seq datasets. The gene lists from the differential expression related to microarray and RNA-Seq analysis were considered Gene Sets 1 and 2, respectively (Supplementary Materials 1 and 2).

### 2.3. Literature Mining to Discover Candidate Genes for Mastitis 

Extensive literature surveys were performed to search for candidate genes using keywords related to bovine mastitis in the PubMed and Google Scholar databases without time limitation. GWAS studies were selected for further detailed review. Then, iHOP (iHOP literature server, http://www.ihop.net.org/ (accessed on 9 October 2021)) was used, which is a web-based tool that allows the exploration of a network of gene and protein interactions by directly navigating the pool of published scientific literature [45]. Finally, the candidate gene list extracted through literature mining was mentioned as Gene Set 3 (Supplementary Material 3).

### 2.4. Determination of Main Gene List

To identify the relevant candidate genes related to mastitis, 3 datasets (microarray, RNA-Seq, and GWAS) from the differential expression and GWAS analyses were integrated. Subsequently, genes that were common to the three gene sets were selected as the main gene list for further analysis. The number of shared DE genes between the 3 datasets was analyzed using the R package VennDiagram v1.6.18 [46].

### 2.5. Functional Enrichment and KEGG Pathway Analysis

Gene ontology (GO) and enrichment analysis were performed using the online programs DAVID [47] (Database for Annotation, Visualization, and Integrated Discovery), PANTHER [48] (Protein ANalysis THrough Evolutionary Relationships), GeneCards (www.genecards.org/ (accessed on 9 October 2021)), g:Profiler [49] (https://biit.cs.ut.ee/gprofiler/gost (accessed on 9 October 2021)), and the STRING database [50] (https://string-db.org (accessed on 9 October 2021)), which are comprehensive web tools that help to explore the biological process (BP), molecular function (MF), and cellular component (CC) of the mined gene set. The pathway enrichment of the identified genes was provided in the Kyoto Encyclopedia of Genes and Genomes (KEGG). Gene Ontology terms with FDR < 0.05 were considered significantly enriched for the identified genes.

### 2.6. Identification of miRNAs and Target Gene Prediction

The functional annotation of the expressed miRNAs consisted of the functional annotation of their potential target genes. The potentially targeted genes were predicted using miRBase [51] (https://www.mirbase.org/ (accessed on 9 October 2021)) and Targetscan [52]. The predicted target genes were selected and submitted to DAVID, KEGG, Reactome pathways, and the PANTHER database for the enrichment target genes of each miRNA.

### 2.7. Reconstruction of Omics Multi-Layers Networks

The miRNA–gene bipartite network was reconstructed based on the master gene list and the molecular interactions documented in related papers and in online interaction databases. Protein–protein interaction (PPI) data were abstracted from the Biomolecular Interaction Network Database (BIND), the Database of Interacting Proteins (DIP), the Biological General Repository for Interaction Datasets (BioGRID), and the Mammalian Protein–Protein Interactions Database (MIPS). Finally, PPI network analysis was performed using the STRING database to explore interactions between genes, specifically in *Bos taurus* species. Each miRNA and target gene was entered into the database and resulting interactions were imported into the networks using Cytoscape software v3.8.2. (National Institute of General Medical Sciences, Bethesda Softworks, Rockville, MD, USA) [53]. Genes and miRNAs in generated networks are represented as nodes and the interactions between these nodes as edges. Furthermore, the metabolic-signaling pathway enrichment of the PPI network was reconstructed using ClueGO v.2.5.5 [54].

### 2.8. Modeling of Three-Dimensional (3D) Structure of Hub Proteins

The SWISS-MODEL template-based approach [55] (https://www.swissmodel.expasy.org/interactive (accessed on 15 October 2021)) was used to predict the 3D structures of hub proteins using individual FASTA sequences and reference PDB files. The resulting PDB files are enclosed in Supplementary Material 4.

## 3. Results

### 3.1. Transcriptome Analysis for Identifying Differentially Expressed Genes (DEGs)

To obtain better insights into the molecular mechanism and the genetic basis of mastitis, we investigated the pattern of transcriptome profiles of mastitis samples versus healthy samples in dairy cattle. The experimental data used for the study were obtained from the GEO database, consisting of microarray and RNA-Seq datasets, as presented in Table 1. The analysis of differentially expressed genes between mastitis and healthy cows was performed based on a fold change > ±2 and a false discovery rate (FDR) < 0.05. The results of the statistical analysis of the microarray datasets showed a total of 564 significant genes from three datasets as follows. The first dataset (GSE93082): 378 DE genes, the second dataset (GSE15020): 177 DE genes, and the third dataset (GSE15022): nine DE genes. Concerning the analysis of RNA-Seq, a total of 774 genes were differentially expressed in the mastitic versus healthy group comparison, of which 442 and 332 genes were detected from accession numbers GSE131607 and GSE75379, respectively. Gene counts and a detailed summary of the alignments for all GEO accession numbers are provided as Gene Sets 1 and 2 for microarray and RNA-Seq analyses, respectively, in Supplementary Materials 1 and 2.

### 3.2. Identification of miRNAs

Based on analysis of the RNA-Seq dataset with access number GSE75379, we identified eight miRNAs out of the 332 DE genes with functions related to mastitis: bta-mir-339a, bta-mir-24-2, bta-mir-222, bta-mir-27a, bta-mir-146a, bta-mir-23a, bta-mir-142, and bta-mir-223. Considering the threshold of a fold change > ±2 and FDR < 0.05, all differentially expressed miRNAs were overexpressed in the mastitic cows compared to the healthy cows (Table 2).

### 3.3. Literature Mining and Identification of Main Gene List

**Figure 2:**
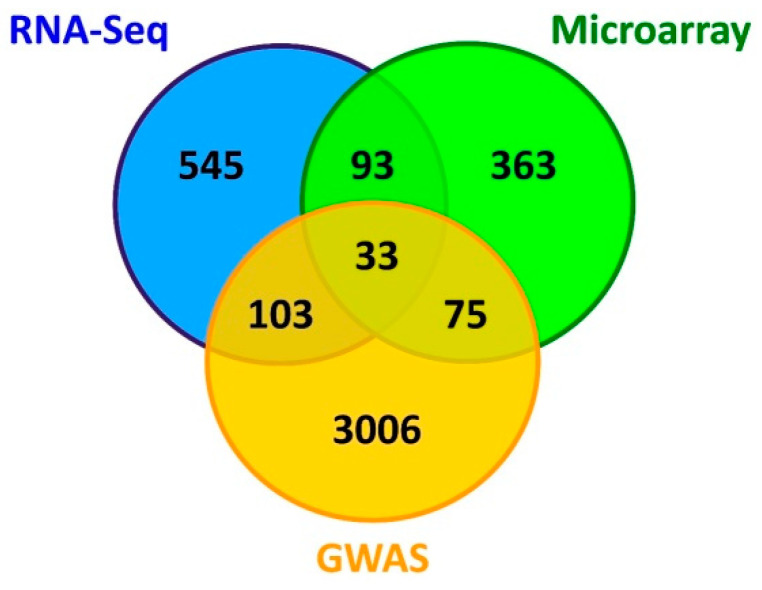
Venn diagram of significant genes among the three types of dataset, including microarray, RNA-Seq, and GWAS data related to mastitis in dairy cattle.

Literature mining was carried out using the iHOP web tool to increase the study’s accuracy and to obtain previous evidence for associations between the identified genes and mastitis in dairy cattle. Based on literature mining, 3217 candidate genes were considered as Gene Set 3 (Supplementary Material 3). The Venn diagram shows the number of genes that are unique or common among the three gene sets for mastitis (Figure 2). Notably, there is a remarkably small overlap between the three datasets. Overall, 33 genes were common in the Gene Sets 1, 2, and 3 relating to microarray, RNA-Seq, and GWAS datasets, respectively, which were named as the main genes involved in mastitis and were considered for subsequent analysis (Table 3). The results of differential expression analysis of 33 common genes based on the RNA-Seq datasets are presented in Table 3. When comparing healthy cows to mastitis cows, of 33 DE genes, nine were underexpressed in the mastitis cows and 24 genes were overexpressed in the mastitis cows, based on their fold-change values (fold change > ±2).

### 3.4. Functional Annotation and Pathway Enrichment Analysis

**Figure 3:**
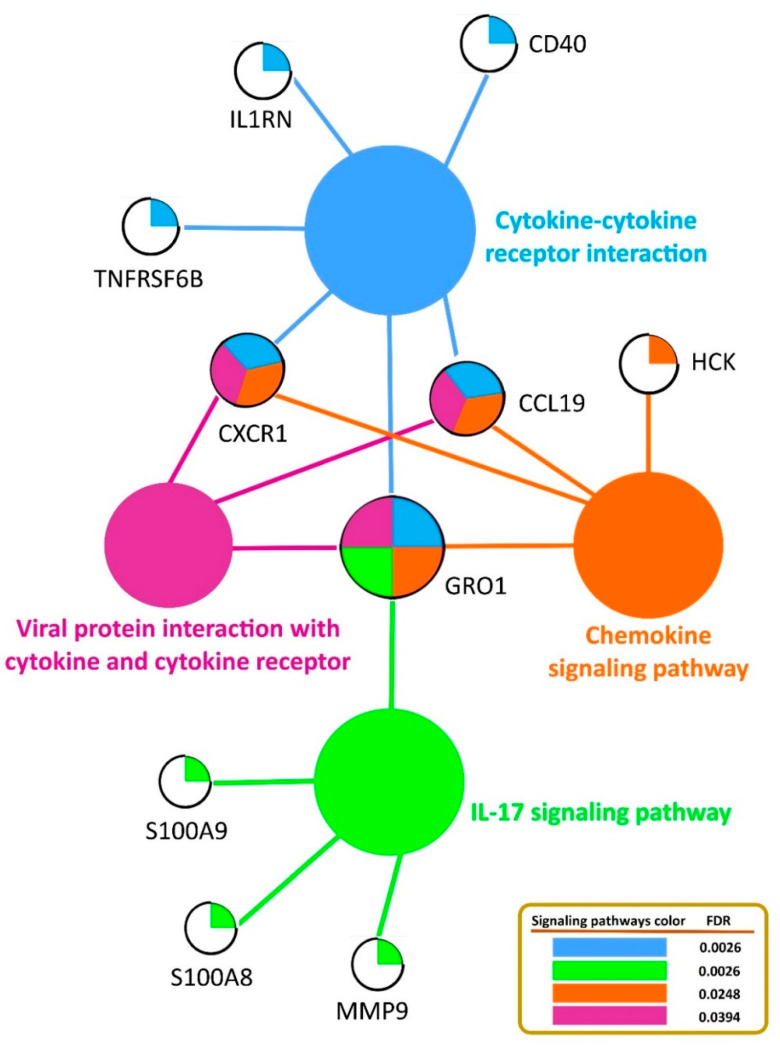
The KEGG pathway-based network analysis of significant genes related to mastitis in dairy cattle.

The functional annotation of GO terms was performed based on the biological process (BP), molecular function (MF), and cellular component (CC) to identify the biological meaning and the systematic features of the list of 33 DE genes, using the DAVID, PANTHER, and g:Profiler databases. Twenty-three biological processes were identified, such as response to stimulus, defense response, response to stress, immune response, cellular process, and biological regulation, which were the most significant ones associated with mastitis. The identified DEGs were significantly involved in the seven following functions: antioxidant activity, binding, protein binding, zymogen binding, arachidonic acid binding, Toll-like receptor 4 binding, and RAGE receptor binding for molecular function. Regarding cellular components, four GO terms, including extracellular region, Golgi lumen, extracellular space, and cellular anatomical entity, were identified (Table 4). In addition, KEGG pathway analysis revealed that the identified DE genes involved in mastitis were enriched in cytokine–cytokine-receptor interaction, the IL-17 signaling pathway, viral protein interaction with cytokines and cytokine receptors, and the chemokine signaling pathway (Figure 3).

### 3.5. PPI Network and Identification of Hub Genes

**Figure 4:**
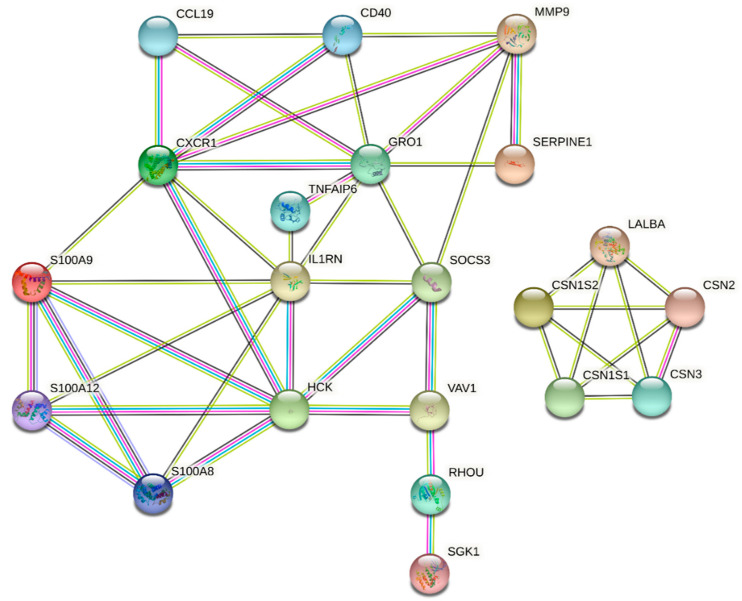
Protein–protein interaction (PPI) network analysis of common differentially expressed genes associated with mastitis in dairy cattle.

Protein–protein interaction (PPI) networks for up- and downregulated genes were reconstructed with the STRING database, which indicated the physical connection between two or more protein molecules related to biochemical functions (Figure 4). Twenty-one nodes with 45 connections (edges) were represented in the PPI network, as presented in Figure 4. Moreover, we considered hub genes based on their higher-degree connectivity values in the PPI network. A total of seven hub genes, including *MMP9*, *HCK*, *GRO1*, *SOCS3*, *CXCR1*, *IL1RN*, and *S100A9*, were identified, all of which were overexpressed genes. All the hub proteins identified are protein-coding genes. The functional enrichment analysis demonstrated that hub genes were involved in the majority of molecular functions and biological processes (Table 4).

### 3.6. Prediction of miRNA-Target Genes and Gene Regulatory Network Reconstruction

**Figure 5:**
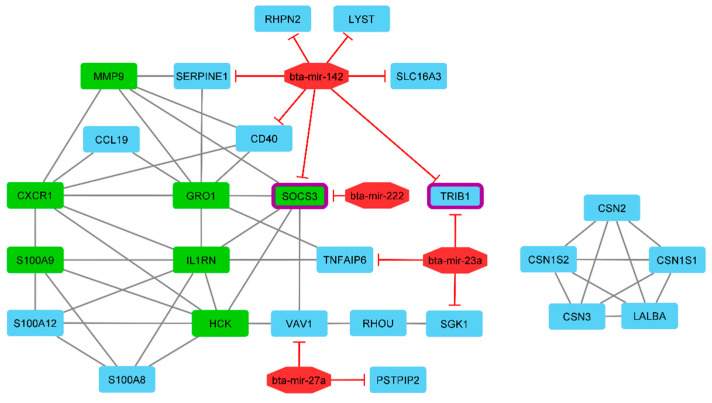
Interactive bipartite network (gene–miRNA) which demonstrates the regulatory effect on mastitis in dairy cattle. In this network, the quadrilateral points represent genes, and the octagonal points represent miRNAs. Regarding miRNAs and target genes, the edges indicate the suppressive role of miRNAs. The edges also represent the gene–gene interactions. The green quadrilateral nodes represent the hub genes. The quadrilateral nodes that have purple around them are the genes showing the highest suppression by miRNAs.

We also aimed to determine whether the expression of miRNAs was associated with that of the 33 DE genes in the mastitic and healthy cows. Among the DE miRNAs, bta-mir-222, bta-mir-27a, bta-mir-23a, and bta-mir-142 suppressed 11 of the identified DE genes as targets of the selected miRNAs. A target gene search using TargetScan demonstrated that bta-mir-142 has seven target genes, namely, *RHPN2*, *LYST*, *SERPINE1*, *CD40*, *SOCS3*, *TRIB1*, and *SLC16A3*, followed by bta-mir-23a having three target genes, *SGK1*, *TNFAIP6*, and *TRIB1*. In addition, bta-mir-27a suppressed the *PSTPIP2* and *VAV1* genes, and bta-mir-222 suppressed the *SOCS3* gene. The *TRIB1* and *SOCS3* genes displayed the highest suppression by miRNAs. The identified target genes, associated with their miRNAs, are visualized in Figure 5. For constructing the gene regulatory network, we compiled a list of DE genes and miRNAs (as nodes) involved in mastitis based on literature mining and PPI resources. Briefly, miRNA–gene bipartite networks are commonly represented in an undirected graph format, with nodes representing miRNAs or genes and edges corresponding to interactions (genes–genes and miRNAs–targeted genes). In this network, we identified 30 nodes (26 genes and four miRNAs), with 57 edges interacting with it (Figure 5).

### 3.7. Three-Dimensional Modeling of Hub Proteins

**Figure 6:**
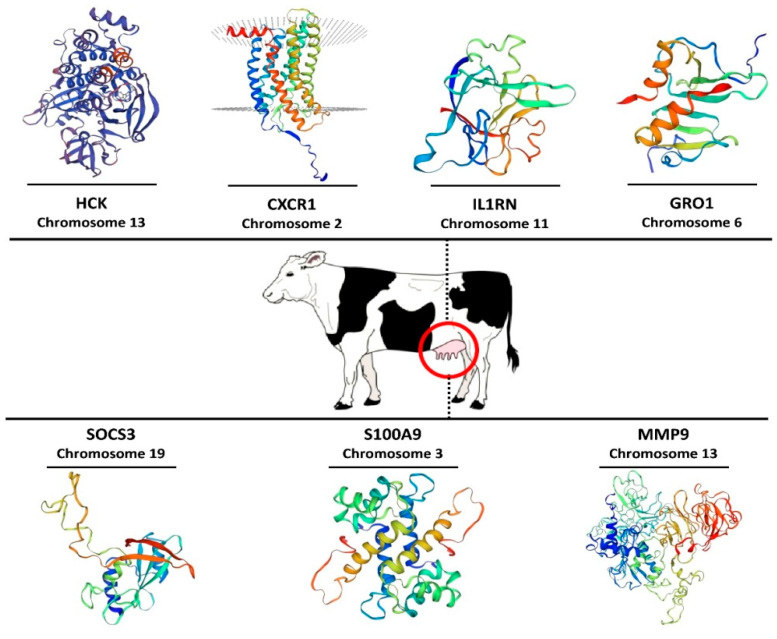
Modeling of three-dimensional protein structure for genes with the most interaction (hub genes) in the interactive bipartite network (gene–miRNA) according to the SWISS-MODEL repository.

In the present study, we also modeled the 3-dimensional protein structure of the seven hub genes identified in the PPI network that had the most interaction with other genes involved in the network (Figure 6). 3D modeling revealed that the predicted structures of these seven hub proteins were significantly different from each other. Four hub proteins (*MMP9*, *HCK*, *CXCR1*, and *S100A9*) had the greatest structural complexity compared to the three other proteins.

## 4. Discussion

Mastitis is a complex trait and is prominent among health-related traits in the cattle industry, exerting a severe impact on profitability and animal welfare. The identification of functional candidate genes and molecular mechanisms involved in mastitis is required, given the persistence of the disease on dairy farms. Moreover, understanding the interplay between molecular and cellular components, with each component interacting at different levels that are entangled in several biological pathways, is important. Hence, the present study provides a general framework to investigate and integrate different sources of transcriptome data and previous results from GWAS studies to identify the genetic basis and key pathways associated with bovine mastitis. Numerous studies have demonstrated that the integration of multiple layers of omics data is a powerful strategy to increase the efficiency and accuracy of candidate gene and biomarker discovery, detecting molecular and biochemical interactions, and the relationships between biological variables in different species [4,7,8,56,57,58]. In this study, the integrative analysis of multiple datasets resulted in prioritizing 33 DE genes as the main gene list, of which nine genes were downregulated and 24 genes were upregulated in the mastitic cows compared with the healthy cows based on their FC values. A list of main detected genes related to mastitis is provided in Table 3, with their main functions (biological process, molecular function, and cellular component) listed in Table 4. Among them, *CSN2*, *CSN3*, *CSN1S1*, *CSN1S2*, *RHPN2*, *LALBA*, *ACSS2*, *RHOU*, and *KRT7* genes were underexpressed in the mastitic cows, mostly located on chromosomes 6. The most important overexpressed genes in cows with mastitis were *GRO1*, *CXCR1*, *SOCS3*, *S100A9*, *MMP9*, *HCK*, and *IL1RN*, which were hub genes (highly connected genes) involved in mastitis in this study. The casein cluster is composed of four genes; β-casein (*CSN2*), κ-casein (*CSN3*), αs1-casein (*CSN1S1*), and αs2-casein (*CSN1S2*), which encode approximately 80% of the protein content of bovine milk [59], and the whey protein gene (*LALBA*) was downregulated in inflamed mammary glands. *LALBA* encodes α-lactalbumin and is essential for lactose synthesis, which plays an important role in milk production as an osmotic regulator of milk secretion [60]. A possible explanation for the lower expression levels of these genes could be that the protein content in mastitic milk would decrease due to an antagonistic genetic relationship between mastitis and protein yield [24]. In previous studies, it was also demonstrated that all five genes were observed in enhanced abundance in the mammary glands of lactating dairy cows [61], dairy sheep [62], and lactating dairy goats [63]. Interestingly, as presented in Table 4, these genes were found in a majority of enriched pathways, suggesting possible key regulatory roles for them. Other noteworthy genes (*RHPN2*, *ACSS2*, *RHOU*, and *KRT7*) showing lower expression in cows with mastitis have critical roles in biological pathways, cellular process, fatty acid synthesis, and metabolism. For instance, *ACSS2* (acyl-CoA synthetase short-chain family member 2) is well known to affect mastitis resistance in dairy cows and plays a role in the activation of acetate for de novo fatty acid synthesis [64]. Similarly to our results, Chen et al. [65] reported lower expression for *ACSS2* and *RHPN2* genes in response to the intramammary infection caused by two different pathogens (*Escherichia coli* and *Streptococcus uberis*) in dairy cows. As presented in Table 3, in the mastitis cows, 24 genes were more highly expressed, of which seven genes were considered as hub genes involved in significantly enriched biological processes and KEGG pathways. Subsequently, the PPI networks and gene regulatory networks were constructed based on these hub genes, which showed significant connectivity and which could shed light on the post-transcriptional regulation of gene expression by the identified miRNAs. Furthermore, the functional enrichment analysis resulted in four significant KEGG pathways associated with mastitis, which comprised six hub genes, i.e., *GRO1*, *CXCR1*, *S100A9*, *MMP9*, *HCK*, and *IL1RN*, as presented in Figure 3. Among these genes, *GRO1* and *CXCR1* were observed in four and three pathways, respectively.

*GRO1* (melanoma growth stimulating activity, alpha) also known as *CXCL1*, is a protein-encoding gene and plays an important role in inflammation and immune defense due to the modulation of leukocyte infiltration [66], which has been previously proposed as a biomarker and therapeutic target in mastitis [67]. This gene is also involved in the metabolic pathways of cytokine–cytokine-receptor interaction, the IL-17 signaling pathway, the chemokine signaling pathway, and viral protein interaction with cytokines and cytokine receptors. In the case of the cytokine–cytokine-receptor interaction pathway, other genes, such as *CD40*, *IL1RN*, *CXCR1*, *TNFRSF6B*, and *CCL19*, were found to be involved in mastitis defense or immune response, as all these genes were upregulated in the mastitic cows based on their FC values. The significant role of cytokines in the immune response to infectious agents is well known because they are soluble extracellular proteins or glycoproteins that are crucial intercellular regulators and mobilizers of cells engaged in innate as well as adaptive inflammatory host defenses, cell growth, differentiation, cell death, and cell development and repair processes. It was previously reported that cytokines can participate in activation of the host defense mechanisms during mastitis [68,69]. The *CXCR1* and *CCL19* genes were also enriched in two other pathways of the chemokine signaling pathway and in viral protein interaction with cytokines and cytokine receptors. *CXCR1* (chemokine (C-X-C motif) receptor 1), identified as a hub gene, is a protein-encoding gene for major pro-inflammatory cytokine receptors [70] that is introduced as a potential genetic marker for resistance to mastitis in dairy cows [71,72]. In our study, the gene *CXCR1* was involved in seven GO terms—response to stimulus, immune response, cellular response to stimulus, neutrophil chemotaxis, biological regulation, cellular process, and cellular response to cytokine stimulus for biological processes (Table 4). In addition, earlier studies have reported that a non-synonymous mutation, c.365C > T, located in exon II of the *CXCR1* gene is associated with susceptibility to mastitis in different breeds of cattle [73,74]. The viral protein interaction with cytokines and the cytokine receptor pathway is an immune system pathway which has a key role in the inflammatory responses to infection and may activate or inhibit cytokine signaling and possibly affect different aspects of immunity. Furthermore, the *S100A8*, *S100A9*, and *MMP9* genes have been recognized as components of the IL-17 signaling pathway. This pathway plays crucial roles in both acute and chronic inflammatory responses. In fact, the interleukin 17 (IL-17) family, as a subset of cytokines, signals via their correspondent receptors and activates downstream pathways that include NF-kappaB, MAPKs, and C/EBPs to induce the expression of antimicrobial peptides, cytokines, and chemokines. *S100A9* and *MMP9*, which were identified as hub genes and which were upregulated in the mastitic cows, play key roles in the regulation of immune response and inflammatory pathways [65,66].

*SOCS3* (suppressor of cytokine signalling 3) was another hub gene identified with higher expression levels, which encodes an intracellular inhibitor of cytokine signaling and has a crucial role in the initial steps of the recognition of a pathogen-associated molecular pattern (PAMP) in the innate immune cells [75]. Furthermore, in the regulatory network, *SOCS3* is suppressed by bta-mir-142 and bta-mir-222. These two miRNAs showed upregulation and their target gene, *SOCS3,* showed the lower expression than them in mastitic cows. We characterized bta-mir-222, bta-mir-27a, bta-mir-23a, and bta-mir-142 as the major miRNAs which play a prominent role in regulating this network of genes and these were upregulated in the mastitic cows. The gene regulatory network showed that the greatest target genes (seven genes) were suppressed by bta-mir-142. There is evidence that miRNAs play a critical role in the regulation of inflammation and immune function during infection with mastitis in dairy cattle [76,77,78,79].

Modeling of the 3D protein structure of hub proteins can be an invaluable aid in order to better understand the details of a particular protein because studies of protein structure and function are becoming a promising approach in the field of bioinformatics. Functional characterization of a protein is often facilitated by its 3D structure. Hence, it is necessary that a 3D structure is determined in examining the proteins’ function at the molecular level. Sequence identity, as a measure of the expected accuracy of a model represented, >30% indicates a relatively good predictor of the model [80]. When sequence identity drops below 30%, the main problem becomes the identification of related templates and their alignment with the sequence to be modeled. Based on our results, among the seven hub proteins, *HCK*, *CXCR1*, *S100A9*, and *MMP9* showed the highest structural complexity, with sequence identities of 94.6%, 75.4%, 69.9%, and 47.5%, respectively, whereas the proteins of *SOCS3*, *IL1RN*, and *GRO1* had the lowest structural complexity with sequence identities of 92.6%, 80.1%, and 72.5%, respectively (Figure 6). Consequently, these findings demonstrate the relevance of integrating results from transcriptomic and functional analyses for a better understanding of the function of important genes and molecular mechanisms responsible for mastitis development.

## 5. Conclusions

The integration of multi-omics data resulted in the identification of 33 common and relevant genes associated with bovine mastitis. Among these, seven genes (*CXCR1*, *HCK*, *IL1RN*, *MMP9*, *S100A9*, *GRO1*, and *SOCS3*) were identified as the hub genes and these can be explored as potential candidate genes for mastitis susceptibility and resistance. Functional annotation and enrichment analysis identified 23, 7, and 4 GO terms related to mastitis in the biological process, molecular function, and cellular component categories, respectively. We identified eight differentially expressed miRNAs, of which four suppressed 11 of the identified genes as their targets. Furthermore, the reconstruction of the regulatory network of genes associated with their miRNAs sheds light on the post-transcriptional regulation of this network. Therefore, this study provides a general framework to investigate and incorporate multiple layers of omics data from high-throughput technologies or available pathway annotation databases, which has led to the elucidation of molecular networks, the cellular and molecular-level features, and the genetic and biological basis of mastitis in dairy cattle.

## Figures and Tables

**Table 1:** Summary of the GEO accession numbers for RNA-Seq and microarray data sets.

No.	Data Type	GEO ^a^ Accession	Platforms	Samples (M:H) ^b^	Citation
1	RNA-Seq	GSE131607	GPL15749 (Illumina HiSeq 2000)	12 (6:6)	Asselstine et al. [16]
2	RNA-Seq	GSE75379	GPL15749 (Illumina HiSeq 2000)	18 (6:12)	Moyes et al. [33]
3	Microarray	GSE93082	GPL2112 ((Bovine) Affymetrix Bovine Genome Array)	12 (6:6)	Zoldan et al. [32]
4	Microarray	GSE15020	GPL2112 ((Bovine) Affymetrix Bovine Genome Array)	10 (5:5)	Mitterhuemer et al. [31]
5	Microarray	GSE15022	GPL2112 ((Bovine) Affymetrix Bovine Genome Array)	10 (5:5)	Mitterhuemer et al. [31]

^a^ GEO, Gene Expression Omnibus; ^b^ M, number of mastitis samples, and H, number of healthy samples.

**Table 2:** Information about differentially expressed miRNAs between the mastitis and healthy samples in dairy cattle based on GSE75379.

miRNA Name	miRNA Region			Fold Change	*p*-Value	FDR
	BTA	miRNA Start	miRNA End			
bta-mir-339a	25	41736134	41736211	2.0472	0.0032	0.0490
bta-mir-24-2	7	11839032	11839103	2.5302	0.0001	0.0056
bta-mir-222	X	98125920	98126030	3.2194	4.88 × 10^−6^	0.0002
bta-mir-27a	7	11838877	11838949	3.6647	1.13 × 10^−8^	1.16 × 10^−6^
bta-mir-146a	7	72071548	72071646	3.9677	2.26 × 10^−8^	2.20 × 10^−6^
bta-mir-23a	7	11838702	11838776	3.9826	8.97 × 10^−9^	9.60 × 10^−7^
bta-mir-142	19	9301432	9301518	4.2675	2.69 × 10^−13^	6.83 × 10^−11^
bta-mir-223	X	94562822	94562929	4.4983	3.05 × 10^−11^	5.58 × 10^−9^

**Table 3:** Summary list of 33 common genes (main genes) in the integrated studies of gene expression and GWAS associated with mastitis in dairy cattle *.

Gene Symbol	Gene Name	Gene Region			Fold Change	*p*-Value	FDR
		Chr	Gene Start	Gene End			
*CSN3*	casein kappa	6	85645854	85658926	−4.1767	8.29 × 10^−10^	1.55 × 10^−6^
*CSN1S2*	casein alpha-S2	6	85529905	85548556	−3.9696	3.53 × 10^−8^	1.85 × 10^−5^
*CSN2*	casein beta	6	85449164	85457744	−3.7916	2.92 × 10^−8^	1.74 × 10^−5^
*RHPN2*	rhophilin Rho GTPase binding protein 2	18	43404074	43474596	−3.4556	1.38 × 10^−6^	0.0002
*CSN1S1*	casein alpha s1	6	85411118	85429268	−3.4530	2.94 × 10^−7^	7.41 × 10^−5^
*LALBA*	lactalbumin alpha	5	31183432	31213145	−3.1099	1.23 × 10^−5^	0.0009
*ACSS2*	acyl-CoA synthetase short chain family member 2	13	64186743	64233568	−2.6729	4.02 × 10^−10^	1.05 × 10^−6^
*RHOU*	ras homolog family member U	28	697339	706882	−2.4960	0.0001	0.0047
*KRT7*	keratin 7	5	27674854	27689030	−2.3804	0.0003	0.0110
*SGK1*	serum/glucocorticoid regulated kinase 1	9	72305979	72418535	2.0107	1.85 × 10^−14^	5.70 × 10^−12^
*TRIB1*	tribbles pseudokinase 1	14	14779050	14787206	2.0650	4.36 × 10^−5^	0.0024
*LYST*	lysosomal trafficking regulator	28	8379173	8523114	2.1066	0.0009	0.0212
*VAV1*	vav guanine nucleotide exchange factor 1	7	17664498	17728163	2.1138	3.31 × 10^−5^	0.0013
*GRO1*	chemokine (C-X-C motif) ligand 1 (melanoma growth stimulating activity, alpha)	6	89072611	89075133	2.2062	0.0003	0.0105
*F5*	coagulation factor V	16	37159073	37238306	2.2729	3.94 × 10^−10^	6.01 × 10^−8^
*SERPINE1*	serpin family E member 1	25	35596139	35617193	2.3498	0.0002	0.0065
*BASP1*	brain abundant membrane attached signal protein 1	20	55908762	55964145	2.4174	3.40 × 10^−8^	3.14 × 10^−6^
*CD40*	CD40 molecule	13	74842191	74853116	2.4243	1.20 × 10^−5^	0.0005
*TNFRSF6B*	TNF receptor superfamily member 6b	13	54054302	54055810	2.4416	0.0001	0.0035
*SLC16A3*	solute carrier family 16 member 3	19	50634317	50642204	2.4821	8.56 × 10^−6^	0.0004
*CXCR1*	chemokine (C-X-C motif) receptor 1	2	106215131	106219158	2.5332	0.0002	0.0065
*SOCS3*	suppressor of cytokine signaling 3	19	53840159	53840858	2.5589	0.0001	0.0047
*CCDC88B*	coiled-coil domain containing 88B	29	42630756	42645750	2.6392	4.24 × 10^−8^	3.81 × 10^−6^
*TNFAIP6*	TNF alpha induced protein 6	2	44747145	44764214	2.7306	0.0001	0.0040
*S100A9*	S100 calcium binding protein A9	3	17115128	17117984	2.9459	5.39 × 10^−6^	0.0005
*PSTPIP2*	proline-serine-threonine phosphatase interacting protein 2	24	45737786	45832060	2.9685	3.43 × 10^−10^	1.05 × 10^−6^
*ALOX5AP*	arachidonate 5-lipoxygenase activating protein	12	30108987	30138259	3.2299	1.64 × 10^−11^	3.14 × 10^−9^
*CCL19*	C-C motif chemokine ligand 19	8	76054024	76055932	3.5366	4.73 × 10^−7^	3.31 × 10^−5^
*MMP9*	matrix metallopeptidase 9	13	74746976	74754303	3.5921	8.64 × 10^−8^	7.35 × 10^−6^
*HCK*	HCK proto-onco, Src family tyrosine kinase	13	61563070	61608503	4.2225	3.23 × 10^−18^	1.69 × 10^−15^
*S100A12*	S100 calcium binding protein A12	3	17102722	17104173	4.4133	4.22 × 10^−11^	7.47 × 10^−9^
*S100A8*	S100 calcium binding protein A8	3	17085577	17086827	4.7179	6.81 × 10^−12^	1.36 × 10^−9^
*IL1RN*	interleukin 1 receptor antagonist	11	46815591	46837831	4.9613	8.55 × 10^−16^	3.36 × 10^−13^

* Information on common differentially expressed genes between the mastitis and healthy samples in dairy cattle provided based on RNA-Seq datasets.

**Table 4:** Top significant gene ontology (GO) terms enriched using genes associated with mastitis in dairy cattle.

Category	Term_ID	Term	Count	FDR	Genes
BP ^1^_DIRECT	GO:0050896	Response to stimulus	20	3.26 × 10^−9^	*CSN2*, *RHPN2*, *SGK1*, *CSN1S2*, *LALBA*, *S100A9*, *CSN1S1*, *SOCS3*, *S100A8*, *ALOX5AP*, *LYST*, *F5*, *RHOU*, *IL1RN*, *MMP9*, *CD40*, *CSN3*, *GRO1*, *S100A12*, *CXCR1*
BP_DIRECT	GO:0032570	Response to progesterone	5	5.21 × 10^−8^	*CSN2*, *CSN1S2*, *LALBA*, *CSN1S1*, *CSN3*
BP_DIRECT	GO:0032355	Response to estradiol	5	3.34 × 10^−7^	*CSN2*, *CSN1S2*, *LALBA*, *CSN1S1*, *CSN3*
BP_DIRECT	GO:0006952	Defense response	9	3.93 × 10^−6^	*CSN1S2*, *LALBA*, *S100A9*, *S100A8*, *LYST*, *IL1RN*, *CD40*, *GRO1*, *S100A12*
BP_DIRECT	GO:0006950	Response to stress	12	7.04 × 10^−6^	*CSN2*, *CSN1S2*, *LALBA*, *S100A9*, *S100A8*, *LYST*, *F5*, *IL1RN*, *MMP9*, *CD40*, *GRO1*, *S100A12*
BP_DIRECT	GO:0006955	Immune response	8	2.06 × 10^−5^	*S100A9*, *S100A8*, *LYST*, *IL1RN*, *CD40*, *GRO1*, *S100A12*, *CXCR1*
BP_DIRECT	GO:0051716	Cellular response to stimulus	14	2.06 × 10^−5^	*CSN2*, *RHPN2*, *SGK1*, *S100A9*, *CSN1S1*, *SOCS3*, *S100A8*, *ALOX5AP*, *RHOU*, *IL1RN*, *MMP9*, *CD40*, *GRO1*, *CXCR1*
BP_DIRECT	GO:0030593	Neutrophil chemotaxis	4	6.46 × 10^−5^	*S100A9*, *S100A8*, *GRO1*, *CXCR1*
BP_DIRECT	GO:0098542	Defense response to other organisms	7	6.46 × 10^−5^	*CSN1S2*, *LALBA*, *S100A9*, *S100A8*, *LYST*, *CD40*, *S100A12*
BP_DIRECT	GO:0006954	Inflammatory response	6	6.92 × 10^−5^	*S100A9*, *S100A8*, *IL1RN*, *CD40*, *GRO1*, *S100A12*
BP_DIRECT	GO:0033993	Response to lipids	6	9.95 × 10^−5^	*CSN2*, *CSN1S2*, *LALBA*, *CSN1S1*, *CSN3*, *GRO1*
BP_DIRECT	GO:0065007	Biological regulation	17	0.00029	*CSN2*, *RHPN2*, *SGK1*, *S100A9*, *CSN1S1*, *SOCS3*, *S100A8*, *ALOX5AP*, *SERPINE1*, *F5*, *RHOU*, *IL1RN*, *MMP9*, *CD40*, *CSN3*, *GRO1*, *CXCR1*
BP_DIRECT	GO:0052548	Regulation of endopeptidase activity	5	0.00063	*CSN2*, *S100A9*, *S100A8*, *SERPINE1*, *MMP9*
BP_DIRECT	GO:0045087	Innate immune response	5	0.0028	*S100A9*, *S100A8*, *LYST*, *CD40*, *S100A12*
BP_DIRECT	GO:0023051	Regulation of signaling	8	0.0041	*CSN2*, *S100A9*, *CSN1S1*, *SOCS3*, *S100A8*, *IL1RN*, *MMP9*, *CD40*
BP_DIRECT	GO:0042981	Regulation of apoptotic process	6	0.0041	*S100A9*, *CSN1S1*, *SOCS3*, *S100A8*, *MMP9*, *CD40*
BP_DIRECT	GO:0050727	Regulation of inflammatory response	4	0.0046	*CSN2*, *S100A9*, *SOCS3*, *S100A8*
BP_DIRECT	GO:0070488	Neutrophil aggregation	2	0.0046	*S100A9*, *S100A8*
BP_DIRECT	GO:0002523	Leukocyte migration involved in inflammatory response	2	0.01	*S100A9*, *S100A8*
BP_DIRECT	GO:0009987	Cellular process	18	0.0104	*CSN2*, *RHPN2*, *SGK1*, *LALBA*, *S100A9*, *CSN1S1*, *SOCS3*, *S100A8*, *ALOX5AP*, *SERPINE1*, *LYST*, *RHOU*, *IL1RN*, *MMP9*, *CD40*, *GRO1*, *S100A12*, *CXCR1*
BP_DIRECT	GO:0032268	Regulation of cellular protein metabolic process	7	0.0221	*CSN2*, *S100A9*, *SOCS3*, *S100A8*, *SERPINE1*, *MMP9*, *CD40*
BP_DIRECT	GO:0050793	Regulation of developmental process	6	0.0276	*CSN2*, *CSN1S1*, *SOCS3*, *RHOU*, *MMP9*, *CD40*
BP_DIRECT	GO:0071345	Cellular response to cytokine stimulus	4	0.0302	*SOCS3*, *CD40*, *GRO1*, *CXCR1*
MF ^2^_DIRECT	GO:0016209	Antioxidant activity	5	2.82 × 10^−5^	*CSN2*, *S100A9*, *CSN1S1*, *S100A8*, *ALOX5AP*
MF_DIRECT	GO:0005488	Binding	19	0.00034	*CSN2*, *SGK1*, *CSN1S2*, *LALBA*, *S100A9*, *CSN1S1*, *SOCS3*, *S100A8*, *ALOX5AP*, *SERPINE1*, *F5*, *RHOU*, *IL1RN*, *MMP9*, *CD40*, *CSN3*, *GRO1*, *S100A12*, *CXCR1*
MF_DIRECT	GO:0005515	Protein binding	14	0.00034	*CSN2*, *CSN1S2*, *LALBA*, *S100A9*, *SOCS3*, *S100A8*, *SERPINE1*, *RHOU*, *IL1RN*, *MMP9*, *CD40*, *CSN3*, *GRO1*, *CXCR1*
MF_DIRECT	GO:0035375	Zymogen binding	3	0.00034	*CSN1S2*, *SERPINE1*, *CSN3*
MF_DIRECT	GO:0050544	Arachidonic acid binding	3	0.00034	*S100A9*, *S100A8*, *ALOX5AP*
MF_DIRECT	GO:0035662	Toll-like receptor 4 binding	2	0.007	*S100A9*, *S100A8*
MF_DIRECT	GO:0050786	RAGE receptor binding	2	0.033	*S100A9*, *S100A8*
CC ^3^_DIRECT	GO:0005576	Extracellular region	13	1.74 × 10^−7^	*CSN2*, *CSN1S2*, *LALBA*, *S100A9*, *CSN1S1*, *S100A8*, *SERPINE1*, *F5*, *IL1RN*, *MMP9*, *CSN3*, *GRO1*, *S100A12*
CC_DIRECT	GO:0005796	Golgi lumen	4	1.38 × 10^−6^	*CSN2*, *CSN1S2*, *CSN1S1*, *CSN3*
CC_DIRECT	GO:0005615	Extracellular space	10	2.57 × 10^−6^	*CSN2*, *CSN1S2*, *LALBA*, *CSN1S1*, *S100A8*, *SERPINE1*, *IL1RN*, *MMP9*, *CSN3*, *GRO1*
CC_DIRECT	GO:0110165	Cellular anatomical entity	22	0.00069	*CSN2*, *RHPN2*, *SGK1*, *CSN1S2*, *LALBA*, *S100A9*, *CSN1S1*, *SOCS3*, *S100A8*, *ALOX5AP*, *SERPINE1*, *KRT7*, *LYST*, *F5*, *RHOU*, *IL1RN*, *MMP9*, *CD40*, *CSN3*, *GRO1*, *S100A12*, *CXCR1*

^1^ BP, biological process; ^2^ MF, molecular function; ^3^ CC, cellular components.

## Data Availability

The data presented in this study are available in Supplementary Materials.

## Supplementary Materials

The following are available online at https://www.mdpi.com/article/10.3390/cimb44010023/s1, Supplementary Material 1: The gene list from the differential expression related to microarray datasets analyses; Supplementary Material 2: The gene list from the differential expression related to RNA-Seq datasets analyses; Supplementary Material 3: The candidate gene list extracted from GWAS results using literature mining; Supplementary Material 4: Predicted structures of the hub proteins in PDB format.

## References

[1] Matukumalli L.K., Lawley C.T., Schnabel R.D., Taylor J.F., Allan M.F., Heaton M.P., O’Connell J., Moore S.S., Smith T.P., Sonstegard T.S. (2009). Development and characterization of a high density SNP genotyping assay for cattle. PLoS ONE.

[2] Meuwissen T.H., Hayes B.J., Goddard M.E. (2001). Prediction of total genetic value using genome-wide dense marker maps. Genetics.

[3] Fondi M., Liò P. (2015). Multi-omics and metabolic modelling pipelines: Challenges and tools for systems microbiology. Microbiol. Res..

[4] Hasin Y., Seldin M., Lusis A. (2017). Multi-omics approaches to disease. Genome Biol..

[5] Ghafouri F., Bahrami A., Sadeghi M., Miraei-Ashtiani S.R., Bakherad M., Barkema H.W., Larose S. (2021). Omics multi-layers networks provide novel mechanistic and functional insights into fat storage and lipid metabolism in poultry. Front. Genet..

[6] Fang L., Sahana G., Su G., Yu Y., Zhang S., Lund M.S., Sørensen P. (2017). Integrating sequence-based GWAS and RNA-Seq provides novel insights into the genetic basis of mastitis and milk production in dairy cattle. Sci. Rep..

[7] Gòdia M., Reverter A., González-Prendes R., Ramayo-Caldas Y., Castelló A., Rodríguez-Gil J.-E., Sánchez A., Clop A. (2020). A systems biology framework integrating GWAS and RNA-seq to shed light on the molecular basis of sperm quality in swine. Genet. Sel. Evol..

[8] Ramayo-Caldas Y., Mármol-Sánchez E., Ballester M., Sánchez J.P., González-Prendes R., Amills M., Quintanilla R. (2019). Integrating genome-wide co-association and gene expression to identify putative regulators and predictors of feed efficiency in pigs. Genet. Sel. Evol..

[9] Kromer J.O., Sorgenfrei O., Klopprogge K., Heinzle E., Wittmann C. (2004). In-depth profiling of lysine-producing Corynebacterium glutamicum by combined analysis of the transcriptome, metabolome, and fluxome. J. Bacteriol..

[10] Sana T.R., Fischer S., Wohlgemuth G., Katrekar A., Jung K.-H., Ronald P.C., Fiehn O. (2010). Metabolomic and transcriptomic analysis of the rice response to the bacterial blight pathogen Xanthomonas oryzae pv. oryzae. Metabolomics.

[11] Yang S., Tschaplinski T.J., Engle N.L., Carroll S.L., Martin S.L., Davison B.H., Palumbo A.V., Rodriguez M., Brown S.D. (2009). Transcriptomic and metabolomic profiling of Zymomonas mobilis during aerobic and anaerobic fermentations. BMC Genom..

[12] Huang S., Chen L., Te R., Qiao J., Wang J., Zhang W. (2013). Complementary iTRAQ proteomics and RNA-seq transcriptomics reveal multiple levels of regulation in response to nitrogen starvation in Synechocystis sp. PCC 6803. Mol. BioSyst..

[13] Kühl I., Miranda M., Atanassov I., Kuznetsova I., Hinze Y., Mourier A., Filipovska A., Larsson N.-G. (2017). Transcriptomic and proteomic landscape of mitochondrial dysfunction reveals secondary coenzyme Q deficiency in mammals. eLife.

[14] Fu F., Cheng V.W., Wu Y., Tang Y., Weiner J.H., Li L. (2013). Comparative proteomic and metabolomic analysis of Staphylococcus warneri SG1 cultured in the presence and absence of butanol. J. Proteome Res..

[15] Ma Q., Zhou J., Zhang W., Meng X., Sun J., Yuan Y.-J. (2011). Integrated proteomic and metabolomic analysis of an artificial microbial community for two-step production of vitamin C. PLoS ONE.

[16] Asselstine V., Miglior F., Suarez-Vega A., Fonseca P., Mallard B., Karrow N., Islas-Trejo A., Medrano J., Cánovas A. (2019). Genetic mechanisms regulating the host response during mastitis. J. Dairy Sci..

[17] Cai Z., Guldbrandtsen B., Lund M.S., Sahana G. (2018). Prioritizing candidate genes post-GWAS using multiple sources of data for mastitis resistance in dairy cattle. BMC Genom..

[18] Kumar N., Manimaran A., Kumaresan A., Jeyakumar S., Sreela L., Mooventhan P., Sivaram M. (2017). Mastitis effects on reproductive performance in dairy cattle: A review. Trop. Anim. Health Prod..

[19] Yang F., Chen F., Li L., Yan L., Badri T., Lv C., Yu D., Zhang M., Jang X., Li J. (2019). Three novel players: PTK2B, SYK, and TNFRSF21 were identified to be involved in the regulation of bovine mastitis susceptibility via GWAS and post-transcriptional analysis. Front. Immunol..

[20] Bakhtiarizadeh M.R., Mirzaei S., Norouzi M., Sheybani N., Vafaei Sadi M.S. (2020). Identification of Gene Modules and Hub Genes Involved in Mastitis Development Using a Systems Biology Approach. Front. Genet..

[21] Oviedo-Boyso J., Valdez-Alarcón J.J., Cajero-Juárez M., Ochoa-Zarzosa A., López-Meza J.E., Bravo-Patino A., Baizabal-Aguirre V.M. (2007). Innate immune response of bovine mammary gland to pathogenic bacteria responsible for mastitis. J. Infect..

[22] Heringstad B., Klemetsdal G., Ruane J. (2000). Selection for mastitis resistance in dairy cattle: A review with focus on the situation in the Nordic countries. Livest. Prod. Sci..

[23] Halasa T., Huijps K., Østerås O., Hogeveen H. (2007). Economic effects of bovine mastitis and mastitis management: A review. Veter. Q..

[24] Heringstad B., Chang Y., Gianola D., Klemetsdal G. (2005). Genetic association between susceptibility to clinical mastitis and protein yield in Norwegian dairy cattle. J. Dairy Sci..

[25] Li N., Richoux R., Boutinaud M., Martin P., Gagnaire V. (2014). Role of somatic cells on dairy processes and products: A review. Dairy Sci. Technol..

[26] Meredith B., Lynn D., Berry D., Kearney F., Bradley D., Finlay E., Fahey A. (2013). A genome-wide association study for somatic cell score using the Illumina high-density bovine beadchip identifies several novel QTL potentially related to mastitis susceptibility. Front. Genet..

[27] Sodeland M., Kent M., Olsen H., Opsal M., Svendsen M., Sehested E., Hayes B., Lien S. (2011). Quantitative trait loci for clinical mastitis on chromosomes 2, 6, 14 and 20 in Norwegian Red cattle. Anim. Genet..

[28] Wagner P., Yin T., Brügemann K., Engel P., Weimann C., Schlez K., König S. (2021). Genome-Wide Associations for Microscopic Differential Somatic Cell Count and Specific Mastitis Pathogens in Holstein Cows in Compost-Bedded Pack and Cubicle Farming Systems. Animals.

[29] Wang X., Ma P., Liu J., Zhang Q., Zhang Y., Ding X., Jiang L., Wang Y., Zhang Y., Sun D. (2015). Genome-wide association study in Chinese Holstein cows reveal two candidate genes for somatic cell score as an indicator for mastitis susceptibility. BMC Genet..

[30] Welderufael B., Løvendahl P., De Koning D.-J., Janss L.L., Fikse W. (2018). Genome-wide association study for susceptibility to and recoverability from mastitis in Danish Holstein cows. Front. Genet..

[31] Mitterhuemer S., Petzl W., Krebs S., Mehne D., Klanner A., Wolf E., Zerbe H., Blum H. (2010). Escherichia coli infection induces distinct local and systemic transcriptome responses in the mammary gland. BMC Genom..

[32] Zoldan K., Schneider J., Moellmer T., Fueldner C., Knauer J., Fuerll M., Starke A., Specht M., Reiche K., Hackermueller J. (2017). Discovery and validation of immunological biomarkers in milk for health monitoring of dairy cows-results from a multiomics approach. J. Adv. Dairy Res..

[33] Moyes K., Sørensen P., Bionaz M. (2016). The impact of intramammary Escherichia coli challenge on liver and mammary transcriptome and cross-talk in dairy cows during early lactation using RNAseq. PLoS ONE.

[34] Du P., Kibbe W.A., Lin S.M. (2008). lumi: A pipeline for processing Illumina microarray. Bioinformatics.

[35] Gautier L., Cope L., Bolstad B., Irizarry R. (2004). Affy-Analysis of Affymetrix GeneChip data at the probe level. Bioinformatics.

[36] Ritchie M.E., Phipson B., Wu D., Hu Y., Law C.W., Shi W., Smyth G.K. (2015). limma powers differential expression analyses for RNA-sequencing and microarray studies. Nucleic Acids Res..

[37] Davis S., Meltzer P.S. (2007). GEOquery: A bridge between the Gene Expression Omnibus (GEO) and BioConductor. Bioinformatics.

[38] Huber W., Carey V.J., Gentleman R., Anders S., Carlson M., Carvalho B.S., Bravo H.C., Davis S., Gatto L., Girke T. (2015). Orchestrating high-throughput genomic analysis with Bioconductor. Nat. Methods.

[39] McInnes L., Healy J., Melville J. (2018). Umap: Uniform manifold approximation and projection for dimension reduction. arXiv.

[40] Andrews S. (2010). FastQC: A quality control tool for high throughput sequence data. Retrieved May.

[41] Bolger A.M., Lohse M., Usadel B. (2014). Trimmomatic: A flexible trimmer for Illumina sequence data. Bioinformatics.

[42] Kim D., Langmead B., Salzberg S.L. (2015). HISAT: A fast spliced aligner with low memory requirements. Nat. Methods.

[43] Liao Y., Smyth G.K., Shi W. (2014). featureCounts: An efficient general purpose program for assigning sequence reads to genomic features. Bioinformatics.

[44] Love M.I., Huber W., Anders S. (2014). Moderated estimation of fold change and dispersion for RNA-seq data with DESeq2. Genome Biol..

[45] Fernandez J.M., Hoffmann R., Valencia A. (2007). iHOP web services. Nucleic Acids Res..

[46] Chen H., Boutros P.C. (2011). VennDiagram: A package for the generation of highly-customizable Venn and Euler diagrams in R. BMC Bioinform..

[47] Sherman B.T., Lempicki R.A. (2009). Systematic and integrative analysis of large gene lists using DAVID bioinformatics resources. Nat. Protoc..

[48] Mi H., Muruganujan A., Thomas P.D. (2012). PANTHER in 2013: Modeling the evolution of gene function, and other gene attributes, in the context of phylogenetic trees. Nucleic Acids Res..

[49] Raudvere U., Kolberg L., Kuzmin I., Arak T., Adler P., Peterson H., Vilo J. (2019). g: Profiler: A web server for functional enrichment analysis and conversions of gene lists (2019 update). Nucleic Acids Res..

[50] Szklarczyk D., Gable A.L., Lyon D., Junge A., Wyder S., Huerta-Cepas J., Simonovic M., Doncheva N.T., Morris J.H., Bork P. (2019). STRING v11: Protein–protein association networks with increased coverage, supporting functional discovery in genome-wide experimental datasets. Nucleic Acids Res..

[51] Kozomara A., Birgaoanu M., Griffiths-Jones S. (2019). miRBase: From microRNA sequences to function. Nucleic Acids Res..

[52] Grimson A., Farh K.K.-H., Johnston W.K., Garrett-Engele P., Lim L.P., Bartel D.P. (2007). MicroRNA targeting specificity in mammals: Determinants beyond seed pairing. Mol. Cell.

[53] Shannon P., Markiel A., Ozier O., Baliga N.S., Wang J.T., Ramage D., Amin N., Schwikowski B., Ideker T. (2003). Cytoscape: A software environment for integrated models of biomolecular interaction networks. Genome Res..

[54] Bindea G., Mlecnik B., Hackl H., Charoentong P., Tosolini M., Kirilovsky A., Fridman W.-H., Pagès F., Trajanoski Z., Galon J. (2009). ClueGO: A Cytoscape plug-in to decipher functionally grouped gene ontology and pathway annotation networks. Bioinformatics.

[55] Waterhouse A., Bertoni M., Bienert S., Studer G., Tauriello G., Gumienny R., Heer F.T., de Beer T.A.P., Rempfer C., Bordoli L. (2018). Swiss-Model: Homology modelling of protein structures and complexes. Nucleic Acids Res..

[56] Backman M., Flenkenthaler F., Blutke A., Dahlhoff M., Ländström E., Renner S., Philippou-Massier J., Krebs S., Rathkolb B., Prehn C. (2019). Multi-omics insights into functional alterations of the liver in insulin-deficient diabetes mellitus. Mol. Metab..

[57] Dao M.C., Sokolovska N., Brazeilles R., Affeldt S., Pelloux V., Prifti E., Chilloux J., Verger E.O., Kayser B.D., Aron-Wisnewsky J. (2019). A data integration multi-omics approach to study calorie restriction-induced changes in insulin sensitivity. Front. Physiol..

[58] Lee B., Zhang S., Poleksic A., Xie L. (2020). Heterogeneous multi-layered network model for omics data integration and analysis. Front. Genet..

[59] Farrell Jr H., Jimenez-Flores R., Bleck G., Brown E., Butler J., Creamer L., Hicks C., Hollar C., Ng-Kwai-Hang K., Swaisgood H. (2004). Nomenclature of the proteins of cows’ milk—Sixth revision. J. Dairy Sci..

[60] Zidi A., Casas E., Amills M., Jordana J., Carrizosa J., Urrutia B., Serradilla J.M. (2014). Genetic variation at the caprine lactalbumin, alpha (LALBA) gene and its association with milk lactose concentration. Anim. Genet..

[61] Seo M., Lee H.-J., Kim K., Caetano-Anolles K., Jeong J.Y., Park S., Oh Y.K., Cho S., Kim H. (2016). Characterizing milk production related genes in Holstein using RNA-seq. Asian-Australas. J. Anim. Sci..

[62] García-Gámez E., Gutiérrez-Gil B., Sahana G., Sánchez J.-P., Bayón Y., Arranz J.-J. (2012). GWA analysis for milk production traits in dairy sheep and genetic support for a QTN influencing milk protein percentage in the LALBA gene. PLoS ONE.

[63] Shi H., Zhu J., Luo J., Cao W., Shi H., Yao D., Li J., Sun Y., Xu H., Yu K. (2015). Genes regulating lipid and protein metabolism are highly expressed in mammary gland of lactating dairy goats. Funct. Integr. Genom..

[64] Bionaz M., Loor J.J. (2008). Gene networks driving bovine milk fat synthesis during the lactation cycle. BMC Genom..

[65] Chen X., Cheng Z., Zhang S., Werling D., Wathes D.C. (2015). Combining genome wide association studies and differential gene expression data analyses identifies candidate genes affecting mastitis caused by two different pathogens in the dairy cow. Open J. Anim. Sci..

[66] Sharifi S., Pakdel A., Ebrahimi M., Reecy J.M., Fazeli Farsani S., Ebrahimie E. (2018). Integration of machine learning and meta-analysis identifies the transcriptomic bio-signature of mastitis disease in cattle. PLoS ONE.

[67] Johnzon C.-F., Artursson K., Söderlund R., Guss B., Rönnberg E., Pejler G. (2016). Mastitis pathogens with high virulence in a mouse model produce a distinct cytokine profile in vivo. Front. Immunol..

[68] Aderem A., Ulevitch R.J. (2000). Toll-like receptors in the induction of the innate immune response. Nature.

[69] Taraktsoglou M., Szalabska U., Magee D.A., Browne J.A., Sweeney T., Gormley E., MacHugh D.E. (2011). Transcriptional profiling of immune genes in bovine monocyte-derived macrophages exposed to bacterial antigens. Vet. Immunol. Immunopathol..

[70] Dinarello C.A. (2000). Proinflammatory cytokines. Chest.

[71] Lahouassa H., Rainard P., Caraty A., Riollet C. (2008). Identification and characterization of a new interleukin-8 receptor in bovine species. Mol. Immunol..

[72] Mao Y., Zhu X., Li R., Chen D., Xin S., Zhu Y., Liao X., Wang X., Zhang H., Yang Z. (2015). Methylation analysis of CXCR1 in mammary gland tissue of cows with mastitis induced by *Staphylococcus aureus*. Genet. Mol. Res..

[73] Pokorska J., Dusza M., Kułaj D., Żukowski K., Makulska J. (2016). Single nucleotide polymorphisms in the CXCR1 gene and its association with clinical mastitis incidence in Polish Holstein-Friesian cows. Genet. Mol. Res..

[74] Zhou L., Wang H., Ju Z., Zhang Y., Huang J., Qi C., Hou M., An L., Zhong J., Wang C. (2013). Association of novel single nucleotide polymorphisms of the CXCR1 gene with the milk performance traits of Chinese native cattle. Genet. Mol. Res..

[75] Strillacci M.G., Frigo E., Schiavini F., Samoré A.B., Canavesi F., Vevey M., Cozzi M.C., Soller M., Lipkin E., Bagnato A. (2014). Genome-wide association study for somatic cell score in Valdostana Red Pied cattle breed using pooled DNA. BMC Genet..

[76] Den Breems N.Y., Nguyen L.K., Kulasiri D. (2014). Integrated signaling pathway and gene expression regulatory model to dissect dynamics of Escherichia coli challenged mammary epithelial cells. Biosystems.

[77] Ju Z., Jiang Q., Liu G., Wang X., Luo G., Zhang Y., Zhang J., Zhong J., Huang J. (2018). Solexa sequencing and custom micro RNA chip reveal repertoire of micro RNA s in mammary gland of bovine suffering from natural infectious mastitis. Anim. Genet..

[78] Lawless N., Foroushani A.B., McCabe M.S., O’Farrelly C., Lynn D.J. (2013). Next generation sequencing reveals the expression of a unique miRNA profile in response to a gram-positive bacterial infection. PLoS ONE.

[79] Li L., Huang J., Zhang X., Ju Z., Qi C., Zhang Y., Li Q., Wang C., Miao W., Zhong J. (2012). One SNP in the 3′-UTR of HMGB1 gene affects the binding of target bta-miR-223 and is involved in mastitis in dairy cattle. Immunogenetics.

[80] Fiser A. (2010). Template-based protein structure modeling. Comput. Biol..

